# Preliminary association analysis of TLR9 gene polymorphisms and immune parameters in an Italian Holstein calves population

**DOI:** 10.1186/1753-6561-5-S4-S20

**Published:** 2011-06-03

**Authors:** Elisabetta Catalani, Alessandro Nardone, Antonino Barone, Maria Teresa Scicluna, Gian Luca Autorino, Andrea Caprioli, Nicola Lacetera

**Affiliations:** 1 Dipartimento di Produzioni Animali, Università degli Studi della Tuscia, Viterbo, 01100, Italy; 2 Istituto Zooprofilattico Sperimentale Lazio e Toscana, Roma, 00178, Italy

## Abstract

**Background:**

This preliminary study was aimed at evaluating the association between single nucleotide polymorphisms (SNPs) on Toll like receptor 9 (TLR9) gene and some immunological parameters in a population of Italian Holstein calves.

**Methods:**

The study was carried out in a commercial farm on 68 Holstein calves aging about 6 months. Genomic DNA was extracted from peripheral blood mononuclear cells (PBMC) and genotyped for nine SNPs on TLR9. Immunological parameters considered were the immunoglobulin (Ig) G titers against bovine herpesvirus 1, and the proliferative response of peripheral blood mononuclear cells to mitogens. For the association study, only results relative to the SNP located in the promoter region have been discussed.

**Results:**

Among the nine SNPs expected, only eight were detected. Considering the SNP located in the promoter region, all three possible genotypes were observed, and their distribution was as follows: genotype a (n=34), b (n=19), and c (n=8). On the basis of their response to vaccine, calves were categorized as low (L, n=8), medium (M, n=45) and high responders (H, n=8). Although no significant association was found between genotypes and L, M or H categories, the genotype estimated as the less represented within the population (c) had no calves categorized as H, the highest frequency of L (25%), and mean values of IgG lower (P < 0.005) compared to genotype b. Furthermore, IgG titers were positively correlated with responses of PBMC to mitogens.

**Conclusions:**

Genotype c appeared to be “non advantageous” in terms of immune response. It was characterized by the presence of the mutation in homozygosity and, not surprisingly, it was the most rare genotype in the population. Larger studies are necessary in order to confirm these observations.

## Background

Bovine herpesvirus 1 (BHV-1) is a virus belonging to herpesviridae family causing disease and immunosuppression in infected cattle [1]. The BHV-1 selectively interferes with the host’s antigen presentation machinery to evade the host’s immune response in vivo [2] and, even when inactivated, is able to induce apoptosis in mitogen-stimulated peripheral blood mononuclear cells (PBMC) in cattle [3]. One of the ways in which immune cells can sense the presence of bacterial and viral DNA is through the Toll like receptor (TLR) 9 [4] that consequently activates not only innate, but also acquired immunity. TLR9 is expressed primarily in antigen-presenting cells such as B cells, where it may facilitate immunoglobulin (Ig) secretion [5]. Within genes involved in immune recognition, several single nucleotide polymorphisms (SNPs) have been described. On bovine TLR9, 17 SNPs were identified [6], and nine of them were observed also in the Holstein breed. In this preliminary study, genomic DNA from an Italian Holstein calves population was genotyped for the nine recognized SNPs on TLR9 to evaluate possible associations with some immunological parameters.

## Methods

The study was carried out in a commercial dairy unit on 68 healthy female Italian Holstein calves aging approximately 6 months at the time of vaccination. Only few of the calves under study were related (common sire). In any case, the analysis of data revealed that such relations were not associated with the ability to mount an immune responses (not shown). Sixty one calves were vaccinated against BHV-1 (Bovilis Ibr, Intervet); the remaining seven calves were not vaccinated and served as negative control. Individual blood samples were taken by the jugular vein 30 days before vaccination, the day of vaccination, and 14, 21, 28, and 42 days post vaccination. Blood samples were used for DNA genotyping, IgG titration, and PBMC separation. Genomic DNA was isolated from whole blood, and genotyping was performed by a private company (Kbioscience, UK). Allele frequencies and deviation from Hardy-Weinberg equilibrium were evaluated using GENEPOP version 3.4 [7]. All blood samples were processed to obtain sera, which were analysed to measure IgG titers against BHV-1 by the serum-neutralization method. On the basis of the mean value of serum IgG recorded after vaccination, calves were categorized as high (H, n=8), medium (M, n=45) or low (L, n=8) responders. The proportion of H, M and L calves was defined according to Begley et al. [8]. Briefly, calves that were 1 standard deviation above the population mean value were categorized as H, whereas those that had residuals 1 standard deviation below the mean value were categorized as L. Blood samples taken 30 days before vaccination and the day of vaccination, were also used to isolate PBMC to perform proliferation tests [9]. Recovery and viability of PBMC were determined by an automated cell counter by the trypan blue exclusion method. The proliferative response of PBMC to mitogens was determined as already described [9]. Mitogens were purchased from Sigma and were the concanavalin A (ConA, 2.5 μg/ml), lipopolysaccharide (LPS, 20 μg/ml**;** from *E. coli* 0111:B4), and pokeweed mitogen (PWM, 1 μg/ml).

Before the analysis, IgG titers in postvaccination sera were log_10_ transformed. Statistical analysis was performed by Anova, and correlations among immunological parameters were calculated by the method of Pearson.

## Results and discussion

One of the nine SNPs expected (723 T/C) was not detected in the population under study. Among the eight SNPs detected, one was in promoter region, two were in intron 1 region and five were located in the exone 2 (one non-synonymous and four synonymous). Allele and genotype frequencies were estimated to be in Hardy-Weinberg equilibrium for all the SNPs observed (data not shown). The association study was carried out considering both the SNP located in the promoter region (-478 A/G) and the non-synonymous SNP on exone 2 (2196 A/G). However, results will be presented only relative to the SNP located in the promoter region, since that on exon 2 did not present any association with the immune parameters. Relative to the SNP located in the promoter region, the three genotypes observed were named a (AG, n=34), b (AA, n=19) and c (GG, n=8).

**Figure 1:**
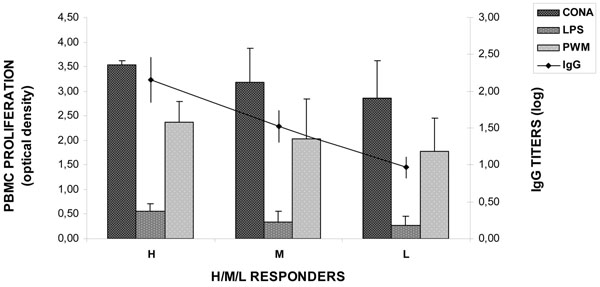
**Mean values of IgG titers against bovine herpesvirus 1 and proliferative response of PBMC stimulated with concanavalin A (CONA), lipopolysaccharide (LPS) or pokeweed mitogen (PWM) in calves categorized as high (H), medium (M) or low (L) responders.** Data are reported as least square means ± standard error.

Although no significant association was found between genotypes and H, M, or L categories, genotype c, the less represented within the population, had no calves categorized as H and the highest frequency of L responders (25%), whereas genotype b had the highest frequency of H (21,05%) and the lowest frequency of L responders (5,3%) (Table 1). Furthermore, calves with genotype c had mean values of IgG titers (1.3±0.21) lower (P < 0.005) than their genotype b counterparts (1.7±0.43). Finally, calves categorized as H, M or L showed a similar response in terms of PBMC proliferation, in that PBMC from H, M or L calves showed also the highest, intermediate and lowest proliferative response to mitogens, respectively (Figure 1). In line with these observations, values of IgG titers were positively (P < 0.05) correlated with those relative to proliferation indicating that in the present study the ability to mount a humoral immune response reflected to some extent that involved in developing a cellular response.

**Table 1:** Distributions of calves according to genotype and type of response to vaccination: high (H), medium (M) and low (L) responders

	***Genotype a (n=34)***		***Genotype b (n=19)***		***Genotype c (n=8)***	
**H/M/L**	n°	%	n°	%	n°	%
H	4	11.76	4	21.05	0	0.00
M	25	73.53	14	73.68	6	75.00
L	5	14.71	1	5.26	2	25.00

## Conclusions

Even if referred to a limited number of animals, these observations testified a certain individual ability of Italian Holstein calves to mount an immune response that may at least partially depend on TLR9 variants and encourage further studies to be carried out on larger populations.

## List of abbreviations used

BHV-1: Bovine herpesvirus 1; ConA: concanavalin A; H: high; Ig: immunoglobulin; LPS: lipopolysaccharides; L: low; M: medium; PBMC: peripheral blood mononuclear cells; PWM: pokeweed mitogen; SNPs: single nucleotide polymorphisms; TLR: toll like receptor

## Competing interests

The authors declare that they have no competing interests.
